# Molecular Investigations of Protriptyline as a Multi-Target Directed Ligand in Alzheimer's Disease

**DOI:** 10.1371/journal.pone.0105196

**Published:** 2014-08-20

**Authors:** Sneha B. Bansode, Asis K. Jana, Kedar B. Batkulwar, Shrikant D. Warkad, Rakesh S. Joshi, Neelanjana Sengupta, Mahesh J. Kulkarni

**Affiliations:** 1 Proteomics Facility, Division of Biochemical Sciences, CSIR-National Chemical Laboratory, Pune, India; 2 Physical Chemistry Division, CSIR-National Chemical Laboratory, Pune, India; Torrey Pines Institute for Molecular Studies, United States of America

## Abstract

Alzheimer's disease (AD) is a complex neurodegenerative disorder involving multiple cellular and molecular processes. The discovery of drug molecules capable of targeting multiple factors involved in AD pathogenesis would greatly facilitate in improving therapeutic strategies. The repositioning of existing non-toxic drugs could dramatically reduce the time and costs involved in developmental and clinical trial stages. In this study, preliminary screening of 140 FDA approved nervous system drugs by docking suggested the viability of the tricyclic group of antidepressants against three major AD targets, viz. Acetylcholinesterase (AChE), β-secretase (BACE-1), and amyloid β (Aβ) aggregation, with one member, protriptyline, showing highest inhibitory activity. Detailed biophysical assays, together with isothermal calorimetry, fluorescence quenching experiments, kinetic studies and atomic force microscopy established the strong inhibitory activity of protriptyline against all three major targets. The molecular basis of inhibition was supported with comprehensive molecular dynamics simulations. Further, the drug inhibited glycation induced amyloid aggregation, another important causal factor in AD progression. This study has led to the discovery of protriptyline as a potent multi target directed ligand and established its viability as a promising candidate for AD treatment.

## Introduction

Alzheimer's disease (AD) is the foremost cause of dementia in the ageing population affecting over 35 million people worldwide. According to World Alzheimer Report 2013, this number is expected to increase by two fold in 2030. AD is a progressive neurodegenerative disorder that leads to the irreversible loss of neurons, intellectual abilities and eventually to death within a decade of diagnosis. Although the molecular bases of AD pathogenesis remains incompletely elucidated, the disease has been recognized as a multifactorial syndrome involving various molecular and cellular processes such as protein aggregation, oxidative stress, cell cycle deregulation and neuroinflammation [1].

There are currently several plausible hypotheses for AD pathogenesis. Cholinergic hypothesis states that the reduced cholinergic neurotransmission leads to the degeneration of cholinergic neurons and hence synaptic failure and cognitive dysfunction [2]. Following this, Acetylcholinesterase (AChE) was validated as a therapeutic target to reduce the degradation of acetylcholine in the synapse. AChE inhibitors (AChEIs) are effective in temporarily restoring cholinergic function, and constitute the majority of AD drugs currently available in the market [3]. However, they are incapable of delaying or preventing neurodegeneration [4], [5].

A variety of biochemical, genetic and pathological studies describe pivotal roles of the Amyloid β (Aβ) peptide in the pathogenesis of AD. “Amyloid hypothesis,” describes the altered synthesis, aggregation and accumulation of Aβ which results in amyloid plaque formation [6]. While extracellular deposits of amyloid plaques are highly neurotoxic, recent studies have also implicated soluble, oligomeric aggregates of Aβ in neurotoxicity [7]. Therefore, a key therapeutic strategy for the treatment of AD involves the development of drugs targeted at inhibiting Aβ production, aggregation, destabilization and clearance of preformed fibrils [8]. A crucial step in Aβ production is the specific N-terminal enzymatic cleavage of the membrane embedded Amyloid Precursor Protein (APP) by the transmembrane aspartyl protease, β-secretase (BACE-1) [9]–[12]. Therefore, inhibiting BACE-1 has been considered as another attractive approach to prevent Aβ neurotoxicity. It is noteworthy that no inhibitors of Aβ aggregation or BACE-1 activity have reached the market yet, despite strong evidence of the causative roles of Aβ in AD.

We further point out that Aβ is transported through a Receptor for Advanced Glycation End products (RAGE) and cause neuronal damage. Long-lived proteins are preferentially modified to form Advanced Glycation End products (AGE), and the stability of Aβ makes it an ideal substrate for non-enzymatic glycation and formation of AGEs [13]. In a recent study, it has been shown that Aβ-AGE formation may intensify the neurotoxicity whereas inhibition of this process significantly rescued the early cognitive deficit in mice [14]. Therefore, glycated Aβ has been considered as a more suitable ligand for RAGE, as it aggravates neuronal deterioration [14]. Hence inhibiting glycation of Aβ may be a valuable therapeutic strategy for AD. However, there has been no concerted effort to explore the inhibition of Aβ glycation as a therapeutic strategy.

Efforts to target AChE inhibition [15]; Aβ production [16]; Aβ aggregation [17]; tau phosphorylation and aggregation [18] have been investigated largely in isolation, despite the complex nature of AD etiology. Recently, drug discovery in AD has gradually inclined towards development of “multi-target-directed ligands” (MTDLs) [19]–[22] which are efficient in treating complex diseases because of their ability to target multiple modes of disease pathogenesis. Further, to evaluate MTDLs for the AD treatment, “drug repositioning” seems to be an appealing strategy, as this approach has several advantages, including reduced time and cost necessary for clinical trials^23^. Priority candidate drugs for hypertension, retinoid therapy, diabetes and antibiotics with sufficient supporting evidences have been considered for repositioning in AD [23]. However, to the best of our knowledge, repositioning drugs for multiple targets in AD is scarce. In this study, an *in silico* screening of 140 FDA approved drugs for neurological treatment was done against the primary targets of AD therapeutics, namely, acetylcholinesterase, BACE-1, and Aβ aggregation. Further i*n vitro* studies showed that amongst selected molecules, the tricyclic antidepressant protriptyline exhibited significant inhibition of AChE, as well as inhibition of other targets of AD. In addition, protriptyline was also found to inhibit glycation mediated Aβ aggregation. Mechanistic insights into protriptyline binding and inhibition of AChE, Aβ, and BACE-1 activity were described in detail with molecular dynamics simulation studies.

## Results and Discussion

### Tricyclic Antidepressant Drugs Display Strong Binding against Various Targets of AD *In silico*

**Figure 1:**
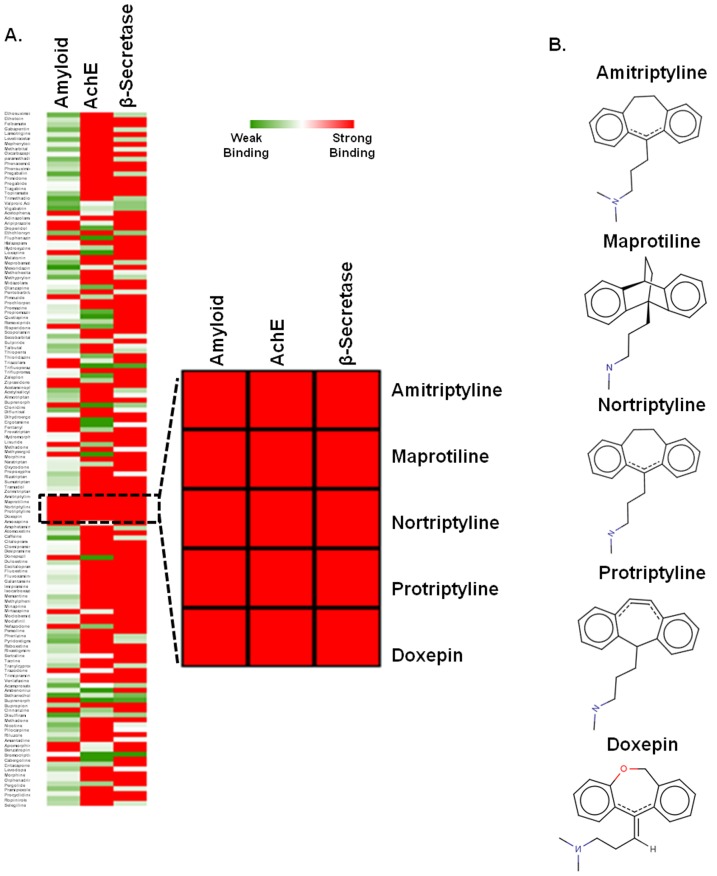
Virtual screening by docking. **A**. Heat map analysis of binding constants of 140 FDA approved nervous system drugs screened against Aβ, AChE and β-secretase by Autodock tool 4.2. In the gradient ruler, red colour indicated strong binding (ΔG<−6 kcal/mol), while green colour indicate weak binding (ΔG>−3 kcal/mol) and the five drugs showing higher affinity to all the above mentioned targets were zoomed. **B**. Chemical structures of the five drugs. All are tricyclic anti-depressant drugs.

Multi-target-directed ligands (MTDLs) are likely to offer promising approaches for treatment of a disease as complex as AD [19]-[21]. The structures of 140 ligands were docked with the major targets of AD viz. AChE, BACE-1 and Aβ aggregation. Ligands were scored based on electrostatic and hydrophobic contributions to the binding energy [24]. Furthermore, polar interactions were considered by H-bonding interactions analysis. Docking scores were used to rank ligands, depending on presence of number of H-bond donors and acceptors within the active sites [25]. Binding energy scores represented in the Heatmap (**Figure 1A**) displayed variability in interactions of the ligands to the three targets of AD. There were several ligands that showed noteworthy interaction with at least two targets, but only few of them had strong interaction with all the targets. It was observed that five antidepressant drugs protriptyline, amytriptyline, maprotiline, doxepin and nortriptyline, which are tricyclic secondary amines showed strong binding affinity and broad specificity toward multiple targets of AD (**Figure 1B**).

### Protriptyline Inhibits AChE Activity by Inducing Conformational Change in the Active Site

**Figure 2:**
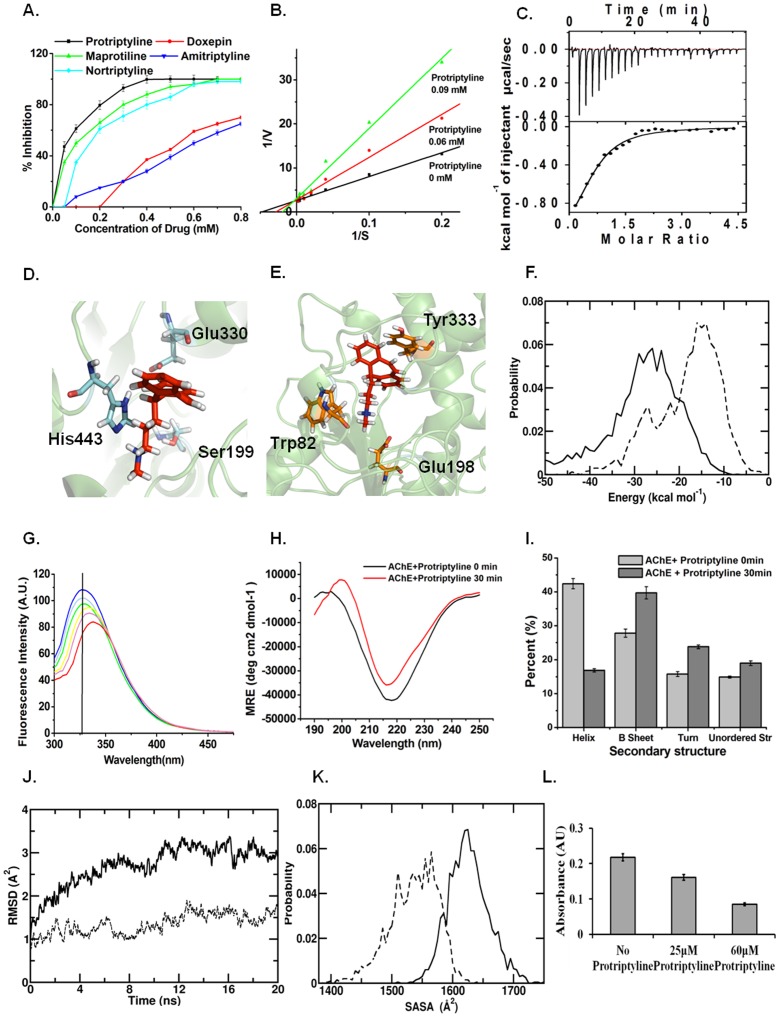
Protriptyline inhibits AChE activity. **A**. Determination of IC50 values of five drugs for AChE by using 0.05–0.8 mM concentration range of all the drugs **B**. Estimation of the kinetic constants by Lineweaver–Burk analysis. AChE inhibition by protriptyline showed competitive inhibition. **C**. Isothermal Titration Calorimetric analysis of protriptyline – AChE interactions. The upper panel shows the raw data in the form of heat effect during titration and the lower panel shows corresponding thermogram representing the best fit curve **D**. Snapshot of drug binding with catalytic subsite of AChE **E**. snapshot of drug binding with anionic subsite of AChE **F**. Distribution of Protriptyline –anionic subsite (solid line) and Protriptyline –esteratic subsite (broken line) nonbonded (nonb) interaction energy; data are averaged over last 20 ns **G**. Fluorescence quenching of AChE by protriptyline **H**. CD spectra of binding of protriptyline to AChE and **I**. CD pro analysis to study the conformational change **J**. Evolution of the backbone RMSD for the Protriptylline bound (solid line) and free (broken line) AChE active sites from MD trajectories **K**. SASA distributions of active sites for Pro-bound (solid line) and free (broken line) AChE active sites from MD trajectories **L**. Measurement of AChE activity after treatment of neuro2a cells with 25 µM and 60 µM protriptyline for 15 h.

AChE currently remains the foremost therapeutic target for AD and the current treatment and management of AD mainly involves use of acetylcholinesterase inhibitors [4], [5]. Therefore, protriptyline, amitryptyline, maprotiline, doxepin and nortriptyline were assessed initially against AChE inhibition. While all the five drugs displayed inhibitory activity against this target (**Figure 2A**), protriptyline displayed highest inhibition, with the least IC_50_ of about 0.06 mM. In comparison, the other ligands displayed relatively higher IC_50_ values; the values corresponding to maprotiline, doxepin, nortriptyline and amitriptyline were 0.1 mM, 0.480 mM, 0.135 mM and 0.6 mM, respectively. Hence, the inhibitory activity of protriptyline against the other targets of AD was evaluated in detail in the remaining study.

The assessment of strong inhibitory activity of protriptyline was consolidated by enzyme kinetic studies, which suggested competitive inhibition of AChE by protriptyline. (**Figure 2B**). The apparent K_m_ for AChE was determined to be ∼0.025 mM by Lineweaver-Burk plot and it was found to be increased in the presence of protriptyline. The inhibition constant K_i_ was determined from Cheng-Prusoff's equation and found to be ∼0.001 mM. Thermodynamic studies of protriptyline-AChE interaction was carried out using Isothermal titration calorimetry (ITC) as it is one of the most widely used quantitative technique for direct measurement of the enthalpy change when two species interact, allowing the determination of heat of association, stoichiometry, and binding affinity from a single experiment [26], [27]. The raw data and corresponding thermogram of the binding experiment is depicted in **Figure 2C**. Binding was strongly exothermic and showed 1∶1 stoichiometry for AChE and protriptyline. The spontaneity of the process is evidenced by a negative change in the enthalpy, ΔH, and a positive change in the entropy, ΔS. Kinetic and ITC data demonstrated the binding of single molecule of protriptyline to the active site of AChE. The active site of AChE comprises two subsites; the anionic subsite (Trp82, Glu198, Tyr333) and the esteratic subsite (Ser199, Glu330 and His443) [28]. Strong binding propensity of protriptyline to both the subsites of AChE was evidenced via the MD studies (**Figures 2D and E**). The mean interaction strengths of protriptyline with the anionic subsite is −31.3 (±9.1) kcal mol^−1^ and −19.4 (±5.0) kcal mol^−1^ with the esteratic subsite (**Figure 2F**). Breakup of the interactions into the non-bonded components showed that electrostatics plays a relatively stronger role than van der Waals interactions in protriptyline binding with active site. The mean interaction strengths of the active site residues with the ligand are provided in **Table S1.**

Protriptylline-AChE interaction was also studied by fluorescence spectrometry. A significant shift in the tryptophan fluorescence emission spectra was observed upon protriptyline binding suggested that the drug induces conformational change in AChE, thereby in its functional abilities. The fluorescence intensity of AChE exhibited an emission maximum at a wavelength, λ_max_, of 325 nm in the unbound state. However, titration of the native enzyme with increasing concentrations of inhibitor resulted in a concentration-dependent quenching of the tryptophanyl fluorescence (**Figure 2G**). A progressive red shift in the emission spectrum with increasing concentration of protriptyline suggested the conformation change in enzyme. Further, the conformational change was confirmed by CD analysis. CD analysis showed that the binding of protriptyline to AChE decreased the minima suggesting protriptyline induces conformational change in AChE. CDPro analysis revealed that binding of protriptyline increased β-sheets and decreased α-helices in AChE. These results are very well in accordance with the fluorescence quenching experiment (**Figure 2H and 2I**).

The evidence of structural distortion was supported by the MD simulation data. In **Figure 2J**, we compare the backbone root mean squared deviation (RMSD) of the active site residues in the free and protriptyline bound state for a sample MD simulation trajectory. The higher RMSD in the latter is a distinct demonstration of the perturbative effect of the ligand on the active site structure. This structural distortion is supported by changes in the inter-residue distances of the active site residues (see **Table S2**). Distinct increases in several of the inter-residue distances are noted, especially in the distances involving Tyr333 and His443. The sharpest increase in the mean inter-residue distance is found for two residues belong to the esteratic subsite, namely Ser199 and His443 that play critical roles in the hydrolysis of acetylcholine. The increase from 7.8 Å in the free state to 11.5 Å in the bound state strongly suggests that protriptyline binding directly affects the catalytic ability of AChE. Further, we found protriptyline binding at the active site to be commensurate with an increase in its solvent accessibility. The solvent accessible surface area, or SASA, was calculated by running a spherical probe of 1.8 Å radius around the surface and calculating the area covered by the probe / calculated in the standard manner. In **Figure 2K**, we have presented histograms of the SASA obtained from the MD trajectories of the free and protriptyline bound AChE. The active site SASA increased from 1533 (±38.9) Å^2^ to 1623 (±33.0) Å^2^ upon protriptyline binding.

Additionally, to confirm the *in vitro* results, the effect of protriptyline on AChE activity was assayed in neuro2a cell line. Protriptyline treated cells showed concentration dependent decrease in AChE activity (**Figure 2L**). These results demonstrated that the *in vitro* IC50 of protriptyline for AChE was sufficient to inhibit its activity in cell culture. Thus, this study suggested that protriptyline inhibits AChE activity *in vitro* as well as in neuro 2a cells, by binding to the active site and causing conformational change.

### Protriptyline Inhibits A*β* Self-Assembly

**Figure 3:**
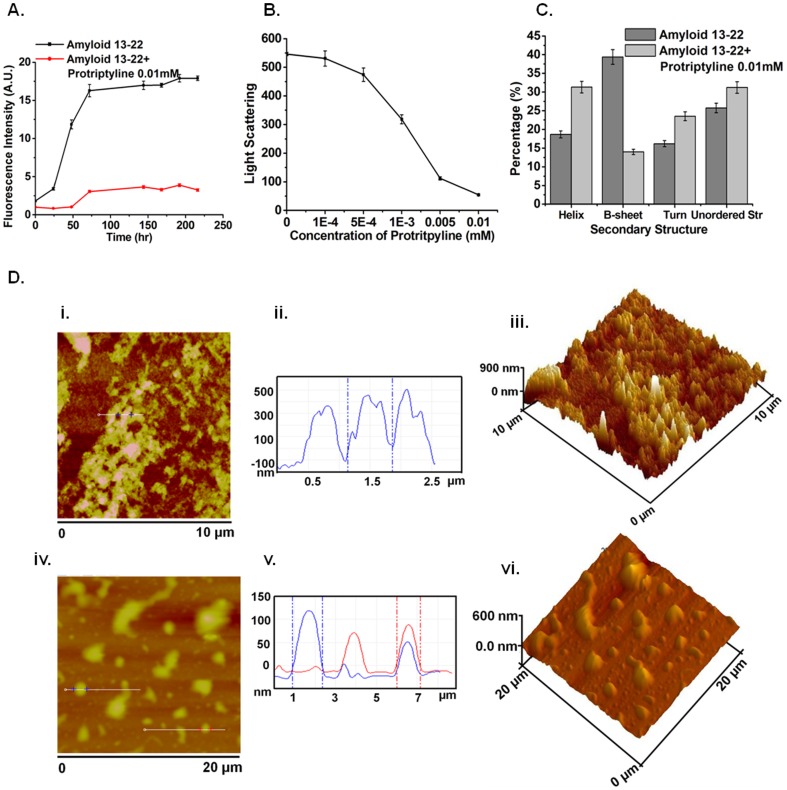
Inhibition of Aβ aggregation by protriptyline. Aβ_13–22_ aggregation in the absence and presence of protriptyline was investigated by **A**. Thioflavin T assay **B**. Light scattering **C**. CD analysis **D**. Atomic Force Microscopy images [i and iv] of aggregated amyloid and Amyloid + protriptyline (0.010 mM) in 10×10 µm^2^ and 20×20 µm^2^ surface area respectively. It is also represented in Line profile [ii and v] and 3D images [iii and vi].

Recent studies suggest that the K_16_LVFF_20_ segment in the Aβ sequence is crucial for the peptide's oligomeric properties as well as fibrillogenetic behavior [29]–[33]. The sequence HHQKLVFFAE corresponding to Aβ_13–22_ was used for our *in vitro* amyloid aggregation inhibition studies. β-sheet rich structures are a common feature of amyloid aggregates [34] and bind to the molecule Thioflavin T (ThT). Therefore, ThT fluorescence assays are frequently used to monitor aggregation of amyloidogenic peptides [35]. In **Figure 3A**, we present ThT fluorescence as a function of time for Aβ_13–22_ in the absence or presence of 10 µM protriptyline. It is observed that the lag time corresponding to the nucleation phase is increased from ∼25 to about ∼50 hours in the presence of protriptyline. The data further shows a dramatic inhibition in the growth phase in the presence of protriptyline. The fluorescence intensity corresponding to the saturation phase was further lowered in the presence of protriptyline, demonstrating the ability of protriptyline to reduce Aβ aggregation. Concentration dependent decrease in Thioflavin T fluorescence was observed in presence of protriptyline at 7^th^ day (**Figure S1**). Static light scattering experiments were performed to investigate the relative decrease in average molecular mass of Aβ aggregates in the presence of protriptyline (**Figure 3B**). As expected, protriptyline reduced the light scattering in a concentration dependent manner. Furthermore, Far-UV CD spectra of the Aβ_13–22_ were recorded with and without protriptyline to monitor any possible alterations in secondary structural propensities (**Figure 3C**). CDPro analysis comparing the spectra obtained at the two conditions showed that protriptyline treatment reduced β-sheet formation and increased overall helicity. The reduced β-sheet formation by CD analysis corroborated the Thioflavin T results. We further performed atomic force microscopy (AFM) experiments on the peptide aggregates formed with and without protriptyline treatment. The results of an AFM scans for Aβ_13–22_ incubated for 7 days with and without protriptyline over scanning areas of 10 µm×10 µm and 20 µm×20 µm are compared in **Figure 3D**.

First, the AFM profiles suggested a distinct reduction in the fibrillar density and a high degree of size dispersion resulting from protriptyline treatment. Further, the line profile and 3D AFM images showed that the average height of the Aβ_13–22_ aggregates was ∼400 nm, with the maximum height reaching ∼600 nm. The height of the aggregates reduced to ∼100 nm in case of protriptyline treated samples. It was quite convincing from the AFM images that the amyloid protein is aggregated and protriptyline treated sample protein showed decreased aggregation.

**Figure 4:**
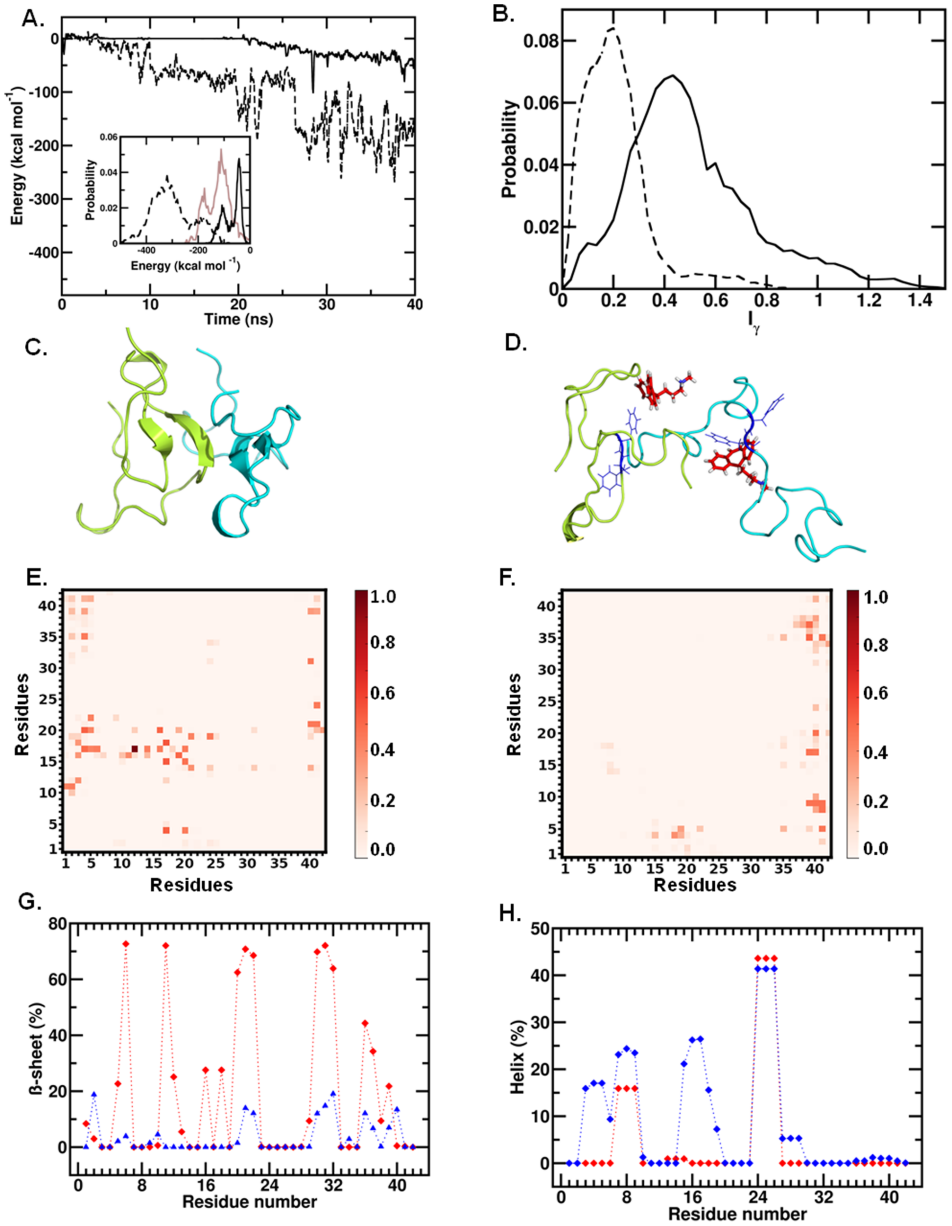
Destabilization of amyloid dimer by protriptyline. **A**. Evolution of monomer-monomer interaction strength over time for free dimer (broken line) and Protriptylline-bound dimer (solid line). *Inset*. Distributions of the interactions from multiple trajectories, and the dimer interactions with Protriptylline (in brown) **B**. Distributions of the asphericity for free (in broken line) and Protriptylline-bound (solid line) dimer **C**. Representative snapshot of most populated cluster of free, and **D**. Protriptylline-bound dimer [16–20 region in blue colour with 19–20 showed in line representation; protriptyline in red colour and two Aβ peptides are in cyan and limon colour respectively] **E**. Residue-residue contact probabilities for free dimer, and **F**. Protriptylline-bound dimer **G**. Residue-wise Beta sheet percentages for free dimer (in red) and Protriptylline-bound dimer (in blue) **H**. Residue-wise helical percentages for free dimer (in red) and Pro-bound dimer (in blue).

For a molecular level insight into the inhibitory action of protriptyline on Aβ self-assembly, we performed MD simulations of Aβ dimerisation in the absence and presence of protriptyline molecules. A β-sheet rich full-length monomer was considered for these studies. A single protriptyline molecule was found to bind to the Aβ monomer with a mean binding strength of −84.0 (±34.0) kcal mol^−1^ (**Figure S2A**). The mean monomer-monomer interaction strength obtained at the end of multiple independent trajectories over a combined total simulation time of 60 ns was −249.1 (±87.1) kcal mol^−1^. In the presence of protriptyline, however, the monomer-monomer interaction weakened significantly, with a mean value of only −69.7 (±31.5) kcal mol^−1^. In **Figure 4A**, we have depicted evolution of monomer-monomer interaction over simulation time for sample simulations in the absence and in the presence of protriptyline; the distributions of these interactions, along with distribution of the protriptyline-dimer interaction is depicted as an inset. The weakening of the inter-monomer interactions due to protriptyline binding results in the formation of complexes that are less compact compared to the pure dimeric forms. In **Figure 4B**, we compare distributions of asphericities I_γ_, of the pure and protriptyline-bound dimer complexes; I_γ_ value of 0.0 denotes perfect sphericity while increasing values denote increased asphericity. The protriptyline bound dimer complexes have a wider distribution and a higher mean value of I_γ_; the mean asphericity values of the free and protriptyline -bound complexes are 0.2 and 0.5, respectively. Further, the mean radii of gyration (R_g_) of these complexes were found to be 13.0 (±0.1) and 16.5 (1.0) Å, respectively. The difference in dimeric compactness upon protriptyline binding is evident from representative snapshots depicted in **Figures 4C and 4D**. Additionally, clustering analysis was done to identify the key residues of Aβ interacting with the protriptyline during molecular dynamics simulation. A representative snapshot from most populated cluster is shown in **Figure 4D**. In two of the most populated clusters (70% of total snapshots used in the clustering analysis), protriptyline interacts most strongly with the KLVFF region.

In order to evaluate the effects of protriptyline binding on the inter-monomer associations, we have analyzed the nature of inter-residue contacts. The average numbers of inter-residue side-chain cross-contacts were reduced up to 46% upon protriptyline binding. Here, as in previous studies [36], we have defined two residues to be in contact if the maximum separating distance of their side-chains does not exceed 7 Å. In **Figures 4E and 4F**, we compare the inter-residue contact probabilities for the free and protriptyline-bound complexes from the MD data. The largest numbers of contacts between the KLVFF regions are lost upon protriptyline binding. However, we note the emergence of a small extent of extraneous contacts, particularly involving the N- and C-terminal regions of the different monomeric units.

Further the effect on the secondary structural propensities of the Aβ units due to protriptyline binding was investigated. Protriptyline was found to reduce β-sheet and induce higher helical propensities in the monomeric form of Aβ (see **Figure S2 B**). **Figure 4G and 4H** present residue-wise β-sheet and helical propensities of the free and protriptyline-bound dimeric complexes, respectively, from the MD data. A sharp decrease in *β*-sheet propensity is found uniformly along the Aβ sequence, including in the residue span H_13_HQKLVFFAE_22_. The decrease in β-sheet propensity is accompanied with an overall increase in helical conformations. Sharp increase in helicity was observed near the N-terminal and KLVFF regions. The alterations to secondary structural propensities thus observed from MD analysis are an excellent corroboration of the CD and ThT binding data.

### Protriptyline Inhibits BACE-1 Activity

**Figure 5:**
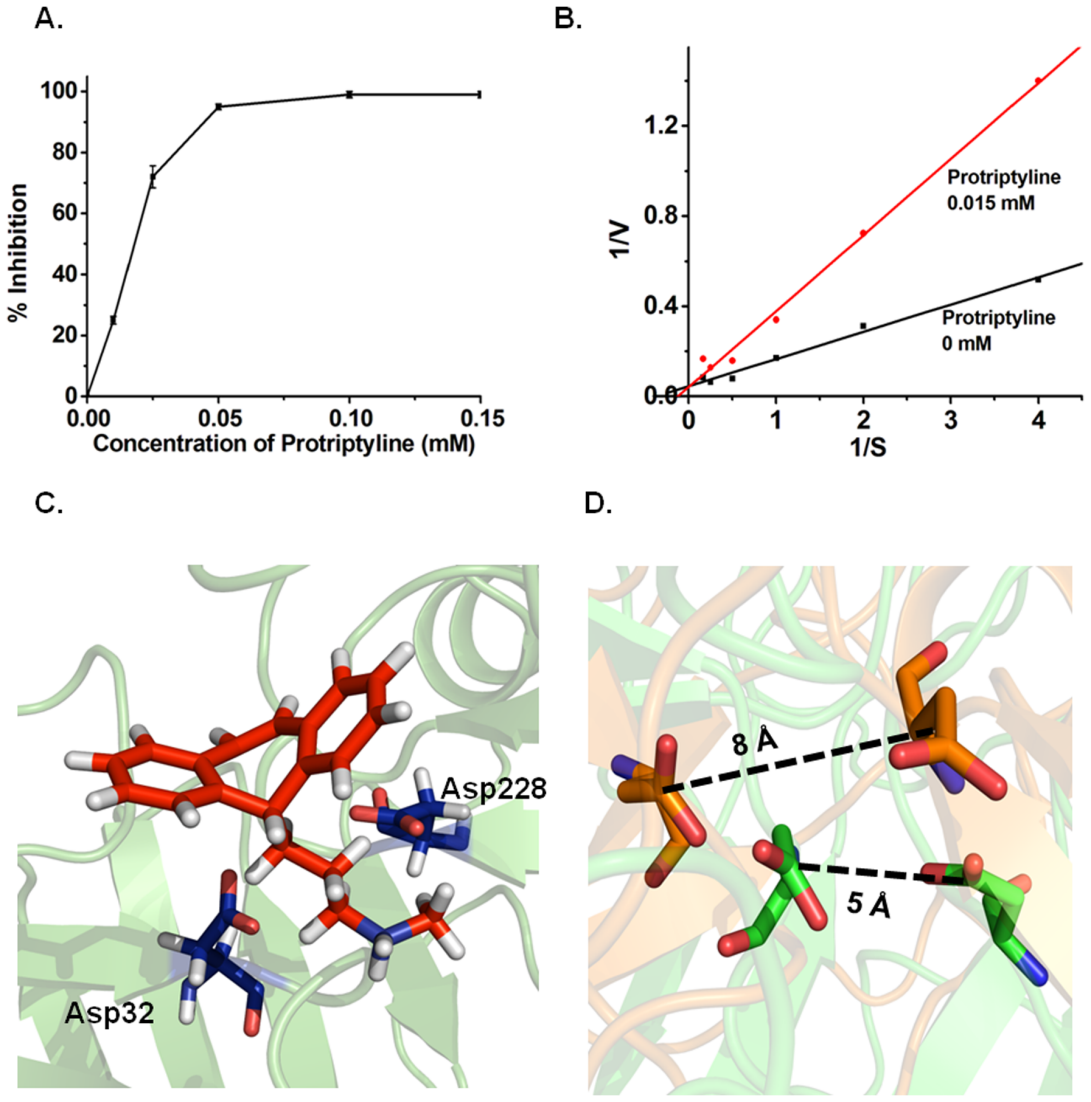
BACE-1 inhibition by protriptyline. **A**. Determination of IC50 of BACE-1 by using various concentrations of protriptyline. The sigmoidal curve indicates the best fit for the percentage inhibition data obtained **B**. Lineweaver-Burk analysis to estimate the kinetic constants. It showed competitive inhibition. **C**. Snapshot of drug binding with active site of BACE-1. Active site residues in BACE-1 are in line representation. **D**. Active site of BACE-1. The structures from unbound (green) and ligand bound (orange) simulations are shown after all - atom superimposition. Snapshots are generated using PyMol.

BACE-1 is a key enzyme required for Aβ production. Hence, BACE-1 inhibition is an attractive target for countering AD [10]–[12]. BACE-1 assay demonstrated decreased activity with increasing concentrations of protriptyline having IC50 ∼0.025 mM (**Figure 5A**). Protriptyline inhibited BACE-1 by competitive inhibition as depicted by Lineweaver-Burk plot (**Figure 5B**). Apparent K_m_ and K_i_ of BACE-1 were calculated as mentioned above for AChE and found to be 0.0025 mM and 0.005 mM respectively. Apparent K_m_ of BACE-1 was increased in the presence of protriptyline Competitive inhibition was evidenced by MD simulation analysis that illustrated protriptyline binds strongly at the active site of BACE-1 comprised of Asp32 and Asp228 (**Figure 5C**). The mean binding strength required for protriptyline to bind to the active site is −29.5 (±7.0) kcal mol^−1^. The center of mass distance between the two residues increases from 5 Å to 8 Å as a result of protriptyline binding (**Figure 5D**). The comparison of the root mean squared deviation (RMSD) of C_α_ atoms of the active site in the unbound state with the protriptyline bound state indicated structural distortion of the arrangement of the active site (see **Figure S3A**). The binding was also found to induce significant alterations to the local secondary structural propensity around the active site (see **Figure S3B**). Therefore, inhibition of BACE-1 by protriptyline is an extra benefit as it prevents Aβ generation. And even if there is some production, protriptyline will obstruct it to get aggregate.

### Protriptyline Inhibits Glycation Associated Aggregation of Aβ

**Figure 6:**
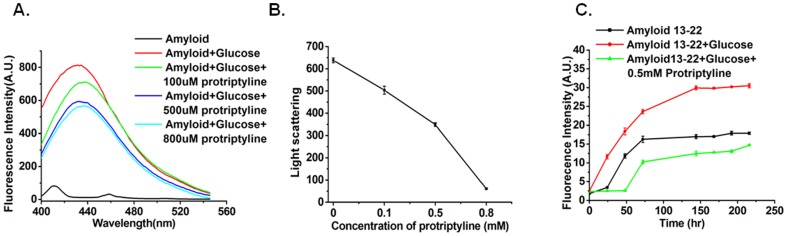
Protriptyline inhibits glycation. **A.** Fluorescence emmission of Aβ and glycated Aβ in presence of various concentration of protriptyline **B**. Light scattering and **C**. Kinetics of amyloid aggregation by Thioflavin T of Aβ_13–22_ and glycated Aβ_13–22_ in the absence and presence of protriptyline.

AD is also referred as type III diabetes [37] and its pathogenesis has been correlated with the extent of glycation [38]. Recent studies suggested glycated Aβ is more neurotoxic than native Aβ [13]; therefore the effect of protriptyline on glycation of Aβ was investigated. Glycated proteins emit fluorescence at 440 nm upon excitation at 370 nm. Fluorescence assay illustrated that Aβ undergoes glycation. The increase in glycation associated fluorescence was reduced by protriptyline in a concentration dependent manner (**Figure 6A**). Glycation enhances the aggregation and also alters the secondary structure of proteins. Static light scattering was used to study glycation induced protein aggregation. Concentration dependent decrease in light scattering was observed (**Figure 6B**). Further, it was studied by Thioflavin T fluorescence assay. Kinetics of aggregation displayed increased Thioflavin T fluorescence during glycation reaction. Lag phase for Aβ and glycated Aβ aggregation was increased in the presence of protriptyline with decreased Thioflavin T fluorescence (**Figure 6C**). In addition to inhibition of Aβ glycation, the drug also inhibited glycation of insulin and BSA(Bovine Serum Albumin) evidenced by MALDI, AGE fluorescence and Thioflavin T fluorescence assay (See **Figure S4 and S5**).

### Protriptyline Does Not Affect Other Proteases

**Figure 7:**
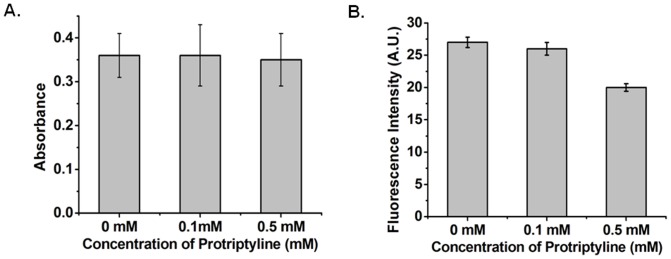
Protriptyline Does Not Affect Other Proteases. Effect of protriptyline on **A**. Trypsin **B**. ADAM 17activity. Specific synthetic substrate BA*p*NA and fluorogenic peptide was used for analyzing activity on trypsin and ADAM 17, respectively. Trypsin activity was unaffected, while ADMA 17 showed weak inhibition in presence of protriptyline.

Protriptyline inhibited multiple targets of AD; therefore we further studied the influence of this drug on other enzymes such as trypsin and α-secretase. It was interesting to observe that it was not able to inhibit trypsin activity even at 0.1 and 0.5 mM (**Figure 7A**). For ADAM 17, protriptyline was found to be a weak inhibitor. It was showing ∼3% inhibition at 0.1 mM and ∼25% inhibition at 0.5 mM (**Figure 7B**). It suggested that protriptyline is not a non-specific inhibitor as it was not able to inhibit other proteases.

### Viability of neuro2a cells in the presence of protriptyline

**Figure 8:**
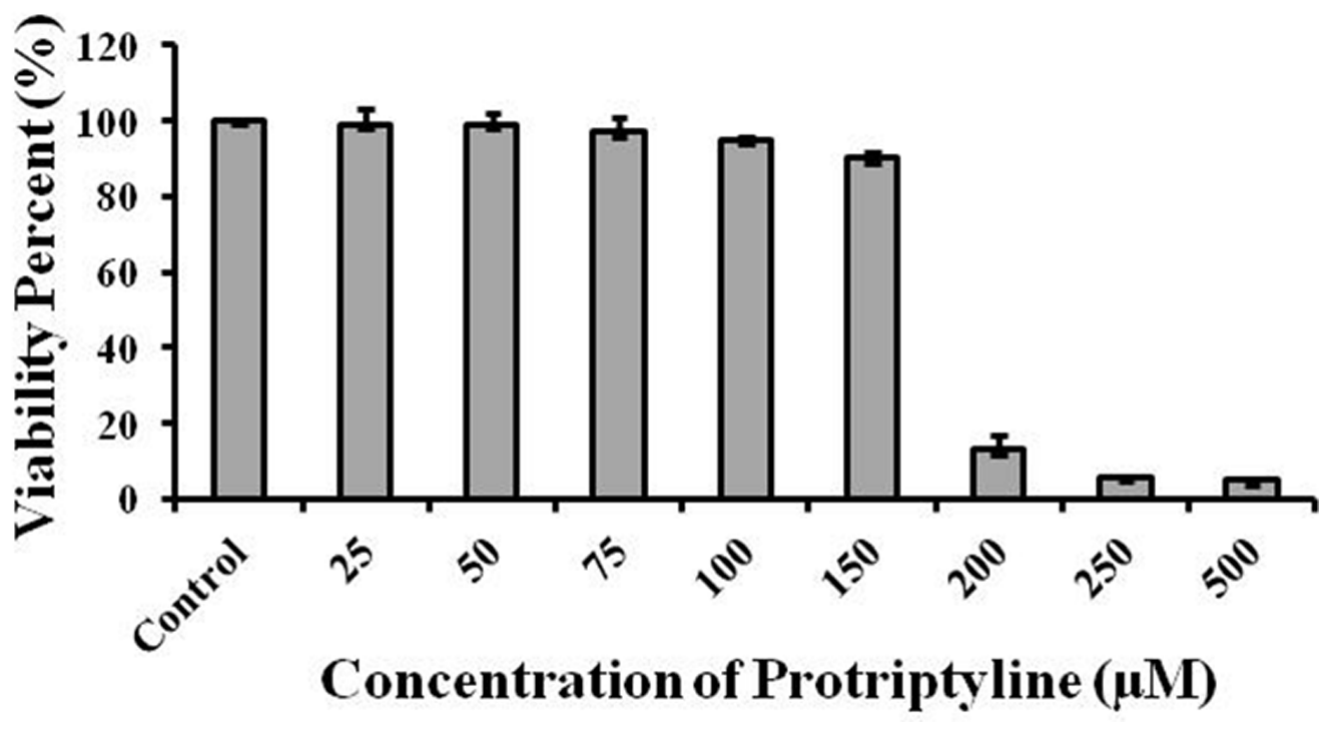
Cell viability in neuro2a cells. Effect of various concentrations of protriptyline (25–500 µM) on cell viability was assessed by MTT assay. Cells were 90% viable up to 150 µM protriptyline concentration.

The effect of various concentrations of protriptyline (25–500 µM) on cell viability was evaluated by MTT assay in neuro2a neuroblastoma cells. It was observed that there was more than 90% of cells were viable upto 150 µM of protriptyline, and a drastic reduction in cell viability was observed at 200–500 µM protriptyline concentrations (**Figure 8**). In this study the IC50 of protriptyline for all the three targets AChE, Aβ, BACE1 was less than 150 µM, this concentration was found to be non toxic to cells.

## Conclusion

**Figure 9:**
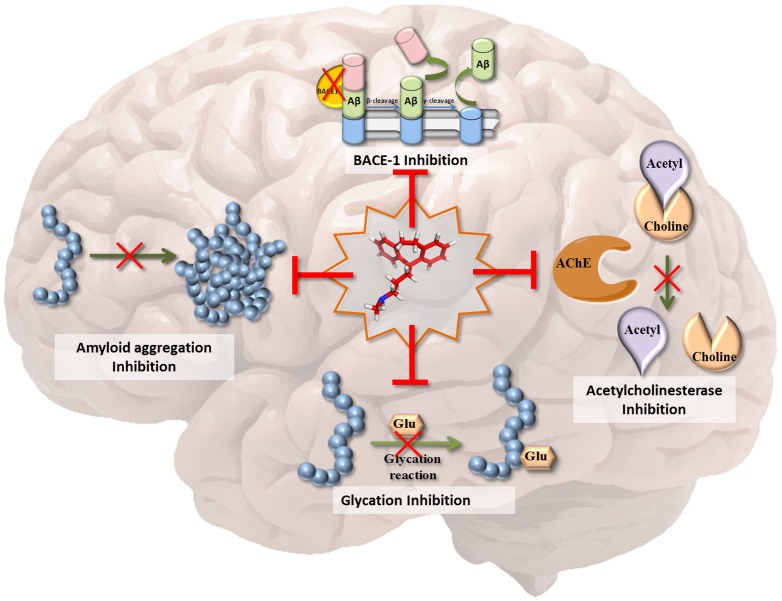
Protriptyline as MTDL. The scheme represents that protriptyline (at the center) is able to inhibit key targets of AD pathogenesis such as AChE, BACE-1, Amyloid aggregation and glycation induced amyloid aggregation.

The multifactorial nature of AD makes its treatment complex and unmanageable. The discovery of molecules that can inhibit multiple pathways of the disease should significantly advance therapeutic strategies. In this study, we investigated the efficacy of the tricyclic antidepressant, protriptyline, against important AD targets. Our *in vitro* and *in silico* investigations established the inhibitory effects of the drug on AChE, amyloid aggregation, BACE-1 and glycation (**Figure 9**). Protriptyline was able to inhibit AChE and β-secretase by binding at the active site and causing conformational changes. In addition, it strongly prevented self-assembly of Aβ and glycated Aβ. It is a FDA approved drug for the treatment of depression, narcolepsy, Attention Deficit Hyperactivity Disorder (ADHD) and headaches and its ability to cross blood brain barrier (BBB) [39] is an additional advantage, which is a crucial requirement of molecules used for intra-cranial diseases. Furthermore, as there is high prevalence rate (30–50%) of AD and depression co-morbidity, the use of antidepressants can be a rational complementary therapy for AD treatment [40]. Therefore, antidepressant activity of this drug could be an added advantage when dealing with AD complications related to depression. To the best of our knowledge, this is the first study in which an anti-depressant drug has been shown to inhibit multiple targets of AD. Our results strongly ratify protriptyline as a promising candidate for AD therapy, and its further evaluation in animal and clinical studies.

## Experimental Section

### Materials

All chemicals were procured from sigma unless and otherwise stated.

### Virtual Screening for Multi-target Drug Ligand for AD

Structures of 140 FDA approved antiepileptics, psycoleptics, analgesics, psycoanaleptics, anti-Parkinson and other nervous system drugs were obtained from DrugBank (The DrugBank database website. Available: http://www.drugbank.ca/. Accessed 2013 Feb 19) database and optimized for their 3D coordinates using Marvin Sketch Tool (ChemAxon website. Available: http://www.chemaxon.com. Accessed 2013 Feb 21). Three dimensional structures of Human acetylcholinesterse (PDB ID: 1B41), β-secretase (PDB ID: 2HM1) and Aβ peptide (PDB ID: 1ZOQ) were accessed from RCSB PDB. Protein structures were energy minimized using Swiss PDB viewer (Swiss-PdbViewer application. Available: http://spdbv.vital-it.ch/. Accessed 2013 Feb 21). AutoDock tool 4.2 [41] was used to convert receptor and ligand from *.pdb to *.pdbqt format and to set other docking parameters. Grid map was set around the active site of acetylcholinesterase (Ser199, Glu330 and His443), β-secretase (Asp32 and Asp228) and KLVFF (residue 17 to 21) region of Aβ protein involved in aggregation. Virtual screening was carried out using AutoDock Vina software and the Lamarckian genetic algorithm as a searching procedure [42]. Binding energy obtained for each complex was represented in heat map format using MeV software packages (MeV: MultiExperiment Viewer. Available: http://www.tm4.org/mev/. Accessed 2013 Mar 5) [43]. The gradient ruler is an indicator of interaction strength. Molecules showing strong binding against all the selected targets were selected for further *in vitro* and molecular simulation studies.

### Acetylcholinesterase Inhibition Assay

The modified method of Ellman et al. [44] was adopted to measure AChE activity. Briefly, 25 µl (0.3 U/ml) AChE from Electric eel fish (*Electrophorus electricus*) was incubated with and without different concentrations of drugs selected from Molecular Docking studies. The reaction was carried out for 15 min at 25°C. 500 µl of 5, 5-dithiobis (2-nitrobenzioc) acid DTNB (3 mM) was then added and reaction was initiated by the addition of 100 µl substrate acetyl thiocholine iodide (ATCI) (15 mM). Total volume of the reaction was made up to 1 ml by Tris buffer, pH 8.0. ATCI hydrolysis was measured by colored product formation, 5-thio-2-nitrobenzoate anion by reaction between DTNB and thiocholine, a hydrolysis product of ATCI. The formation of the colored product was measured at 405 nm wavelength after 30 min. The background was determined from negative controls (omission of AChE enzyme).

### Isothermal Titration Calorimetry

ITC was performed using Microcal Auto-iTC instrument (GE Healthcare). 40 injections of 2 µl protriptyline (Stock  = 2.2 mM) was titrated against 0.3 U/µl solution of AChE. Experiments were carried out at 25°C in a Tris buffer, pH 8.0. Reference titration was carried out by injecting the same concentration of protriptyline into buffer. Reference titration was subtracted from experimental titration. Origin 6.0 software was used to derive affinity constants (K_d_), the molar reaction enthalpy (ΔH) and the stoichiometry of binding (N), by fitting the integrated titration peaks.

### Fluorescence Analysis of AchE-Protriptyline Interaction

AchE-protriptyline interaction was also studied by measuring tryptophan fluorescence using Varioscan plate reader. AChE was excited at 280 nm and emission was scanned from 300 nm to 500 nm. Titration of enzyme with protriptyline was performed by the addition of different concentrations of inhibitor (10 µM–100 µM) to a fixed concentration (0.2 U/µl) of enzyme solution. Background buffer and inhibitor spectra were subtracted and graphs were smoothed.

### BACE-1 Inhibition Assay

BACE-1 activity was studied in accordance with the manufacturer's instructions (Sigma). To test the effect of protriptyline on BACE-1 activity, 1.8 U of BACE-1 enzyme and 10 µM of β-secretase specific peptides conjugated to fluorogenic reporter molecules were incubated with or without various concentrations of protriptyline for 2 h at 37°C. Negative control included all the reactants except BACE-1 enzyme. After 2 h, fluorescence emission was measured at 405 nm upon excitation at 320 nm.

### Inhibition Kinetics for AChE and BACE-1

Michaelis–Menten constant (K_m_) was determined by measuring the activities of AChE and BACE-1 using various concentrations of ATCI (100 µM–1000 µM) and BACE-1 substrate (0.25 µM–6 µM) respectively. Lineweaver-Burk double reciprocal plot was plotted in order to determine the K_m_. The protriptyline inhibition kinetics was analyzed over a range of concentration (50 µM–1500 µM) and (10 µM–150 µM) for AChE and BACE-1 respectively. The IC_50_ of protriptyline for both the enzymes was calculated by determining the inhibitor concentration at which the enzyme activity is 50% inhibited. The K_i_ was calculated directly from IC_50_ value using Cheng-Prussoffs classical equation [45]. 
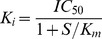



In order to determine the type of inhibition, AChE and BACE-1 were incubated with 60 µM and 15 µM protriptyline concentrations respectively and assayed at increasing concentrations of AChE substrate (ATCI, 100 µM–1000 µM) and BACE-1 substrate (1 µM–10 µM) respectively. The reciprocals of reaction rate (1/v) for each inhibitor concentration were plotted against the reciprocals of the substrate concentrations (1/S). Mode of inhibition by protriptyline was determined from the graphical representation.

### Inhibition of Aβ Aggregation

Aβ_13–22_ peptide (HHQKLVFFAE), the aggregation prone region of Aβ, was synthesized from Thermo Fisher Scientific. Synthetic Aβ_13–22_ peptide was dissolved in 10% ammonium hydroxide and sonicated for 5 min. It was then diluted in 10 mM PBS, pH 7.0 to a final concentration of 200 µM. 100 µM of Aβ_13–22_ was incubated with and without various concentrations of protriptyline in 10 mM PBS, pH 7.0 at 37°C for a week and these samples were further used for aggregation inhibition assays.

### Glycation Inhibition Assay

Anti-glycation activity of protriptyline was studied using insulin, BSA and Aβ. The details of insulin and BSA glycation are described in **Method S1**. In Aβ glycation studies, 100 µM Aβ_13–22_ and 0.1 M glucose were incubated with and without various concentrations of protriptyline in 10 mM PBS, pH 7.0 for 7 days. These samples were further used to study glycation mediated Aβ aggregation by different assays.

### Thioflavin T Assay

Thioflavin T assay was performed on 7^th^ day of incubation. 50 µl (25 µM) of Aβ_13–22_ was mixed with 150 µl of Thioflavin T (ThT) stock solution (50 µM ThT in PBS pH 7.0) and placed in 96-well plate (black with flat bottom, Cornings). Fluorescence emission was measured at 460–550 nm upon excitation at 440 nm. To account for background fluorescence, the fluorescence intensity measured from each control solution without Aβ was subtracted from each solution containing Aβ_13–22_. Similarly, Thoflavin T assay was performed for aggregation kinetics of Aβ_13–22_ and Aβ_13–22_ glycation with and without protriptyline.

### Light Scattering Analysis

Inhibition of aggregation/ glycation mediated aggregation of Aβ was detected by static light scattering method using a Perkin-Elmer Luminescence spectrometer LS50B. Both excitation and emission wavelengths were set at 400 nm. Excitation and emission slit width was set to 10 nm and 2.5 nm, respectively. Scattering was recorded for 60 sec.

### Circular Dichroism Spectroscopy

The far UV CD spectra (in wavelength range of 190–250 nm) of Aβ13–22 (20 µg/ml) with and without protriptyline was recorded on a Jasco-J815 spectropolarimeter at ambient temperature. In case of AChE, the enzyme was incubated with protriptyline and CD spectra were recorded at 0 min and 30 min. Each CD spectrum was accumulated from three scans at 50 nm/min with cell path length of 0.1 cm. Contribution due to buffer was corrected in all spectra and observed values were converted to mean residual ellipticity (MRE) in deg cm^2^ dmol^−1^ defined as 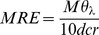



Where M is the molecular weight of the protein, θ_λ_ is CD in millidegree, d is the path length in cm, c is the protein concentration in mg/ml and r is the number of amino acid residues in the protein. Secondary structure content of the amyloid with and without protriptyline was calculated using the CDPro software (CDPro software package. Available: http://lamar.colostate.edu/~sreeram/CDPro/main.html. Accessed 2013 Oct 18).

### Atomic Force Microscopy

For atomic force microscopy (AFM) analysis, 10 µl of each sample was deposited on a piece of freshly cleaved mica disk. The disk was washed with water and dried overnight. The sample was mounted onto a Multimode scanning probe microscope equipped with a Nanoscope IV controller from Veeco Instrument Inc., Santa Barbara, CA. All the AFM measurements were done under ambient conditions using the tapping-mode AFM probes model - Tap190Al purchased from Budget Sensors. The radii of tips used in this study were less than 10 nm, and their height was ∼17 µm. The cantilever used had a resonant frequency of ca. 162 kHz and nominal spring constant of ca. 48 N/m with a 30 nm thick aluminium reflex coating on the back side of the cantilever of the length 225 µm. For each sample, three locations with a surface area of 20×20 µm^2^ and 10×10 µm^2^ for amyloid and protriptyline treated amyloid were imaged with a frequency of 1 Hz and at a resolution of 512×512 dpi. Representative images were selected for comparative studies.

### Measurement of Glycation Associated Fluorescence

Glycation associated fluorescence of Aβ was measured in amyloid, glycated amyloid treated with or without protryptiline at 370 nm excitation and emission was scanned from 400–550 nm.

### BA*p*NA Assay

Activity of Bovine trypsin was estimated using enzyme-specific chromogenic substrate BA*p*NA [46]. In brief, 10 µg Bovine trypsin was incubated with and without 100 µM, 200 µM, 500 µM protriptyline at 37°C for 15 min and volume was made upto 150 µl with 0.1 M Tris-HCl pH 7.8. Further, 1 ml BA*p*NA was added to the reaction mixture and incubated for 10 min at 37°C. The reaction was terminated by addition of 200 µl of 30% acetic acid and absorbance was measured at 410 nm.

### ADAM17 Assay

ADAM17 assay kit (Enzo Life Sciences) was used to study the effect of protriptyline on ADAM17. Briefly, ADAM17 and fluorogenic peptide (substrate) was incubated with and without 100 µM protriptyline at 37°C for 10 min. The negative controls had the reaction mixture without enzyme. Fluorescence was measured at 328 nm and 420 nm for excitation and emission respectively.

### Cell culture

Murine neuro2a neuroblastoma cells were obtained from National Center for Cell Science, Pune, India. The cells were maintained in Dulbecco's modified Eagle's medium (DMEM) and 10% Fetal Bovine Serum (FBS). Cells were maintained at 37°C in humidified air containing 5% CO_2_ and were grown in monolayer cultures.

### Determination of AChE inhibition in cultured cells

Equal numbers of cells were seeded in 25 cm^2^ flasks (1.5 million). Cells were allowed to adhere and attain their morphology. Cells were serum starved for 24 h and treated with or without 25 µM or 60 µM of protriptyline concentration for 16 h. Cells were trypsinised and given two washes with ice cold PBS. Protein was extracted by sonicating the cells for 20 min. The cell lysate was centrifuged for 60 min at 16000 rpm. Supernatant was collected and protein concentration was determined by Bradford's method. Acetylcholinesterase activity was assayed as described above by Ellman's assay.

### Cell viability

Cell viability following exposure to protriptyline was measured by MTT reduction assay. Neuro 2a (N2a) neuroblastoma cells were seeded at a cell density of 1×10^4^ cells per well in a 96 well plate. After the cells adhered and attained their morphology, they were serum starved for 24 h prior to treatment with various concentrations (25–500 µM) of protriptyline in triplicate for 15 h. After incubation, cells were given one wash with PBS and 100 µl fresh serum free media was added. 20 µl of 5 mg/ml MTT (dissolved in PBS) was added to each well and incubated in dark at 37°C until violet formazan crystals were observed. Media from each well was discarded and crystals were dissolved in 100 µl DMSO. Absorbance was measured at 555 nm using Biorad iMark microplate reader.

### Molecular Dynamics Simulations

All simulations in this study were carried out with the NAMD2.9 package [47], using the CHARMM22 all-atom force field with CMAP correction for the proteins [48], [49]. Force field parameters for protriptyline were generated using the SwissPARAM tool [50], and refined via electronic structure calculations using Gaussian03. This strategy has been used in several recent studies [51]–[53]. Simulations were carried using a time step of 2 fs in the isothermal-isobaric (NPT) ensemble at a temperature of 310 K and a pressure of 1 atmosphere. Each system was sampled for a total duration of 60 ns with multiple trajectories. The SHAKE algorithm [54] was used to constrain bond lengths involving hydrogen atoms. Constant temperature was maintained with Langevin dynamics with a collision frequency of 1 ps^−1^, and constant pressure was maintained using the Langevin piston Nose-Hoover method [55]. Three-dimensional orthorhombic periodic boundary conditions were employed and full electrostatics calculated with the particle-mesh Ewald method [56]. A non-bonded cutoff distance of 12 Å was employed, which were smoothened at a distance of 10.5 Å. Details of system setups and trajectory analysis are provided in **Method S2**.

### Statistical Analysis

All the experiments performed independently three times. Student's t-test was used for statistical analysis. Data were expressed as mean ±SD. A *p*-value <0.05 was considered as statistically significant.

## Supporting Information

Figure S1 **Thioflavin T Assay.** Protriptyline causes concentration dependent decrease in Aβ aggregation.
(DOCX)

Figure S2 **A. Snapshot of drug binding with Aβ monomer.** Central hydrophobic core are in line representation **B. Comparison of residue wise percentage of helix from unbound and ligand bound Aβ monomer simulated trajectory.**
(DOCX)

Figure S3 **A. Root mean square deviation (RMSD) of active site regions of β-secretase in unbound (red) and ligand bound (blue) simulated trajectory. B. Comparison of residue wise percentage of beta from unbound (red) and ligand bound (blue) simulated trajectory.**
(DOCX)

Figure S4 **A. Control insulin B. Glycated insulin C. glycation inhibition in presence of 500 µM and D. 1000 µM protriptyline.** These spectra were acquired on a positive reflector mode by MALDI-TOF-MS. Glycated peaks are shown by black arrow.
(DOCX)

Figure S5 **BSA (Bovine Serum Albumin) glycation inhibition assay.** Glycation inhibition of BSA was studied by A. Measurement of AGE fluorescence, % glycation inhibition was plotted and B. Thioflavin T fluorescence assay, concentration dependent decrease in thioflavin T fluorescence was observed.
(DOCX)

Table S1 **Acetylcholinesterase-protriptyline interaction energy calculations.** Interaction energy of ligand with active site residues averaged over last 20 ns of all simulated trajectories. Interaction strengths are in kcal mol-1 unit. Standard deviations are provided within braces.
(DOCX)

Table S2 **Inter-residue distance between active site of AChE residues averaged over last 20 ns of free (vertical) and ligand bound (horizontal) simulated trajectories.** Distances are in Å unit. Standard deviations are provided within braces.
(DOCX)

Method S1 **Inhibition of Insulin and BSA glycation.**
(DOCX)

Method S2 **System Setup for Molecular Dynamics Simulation.**
(DOCX)
