# Supplementary material for: Molecular Investigations of Protriptyline as a Multi-Target Directed Ligand in Alzheimer's Disease
Source: PLoS One. 2014 Aug 20;9(8):e105196. doi: 10.1371/journal.pone.0105196 (PMC4139341; doi:10.1371/journal.pone.0105196)

**Supplementary Figure S1.** Thioflavin T Assay. Protriptyline causes concentration dependent decrease in Aβ aggregation


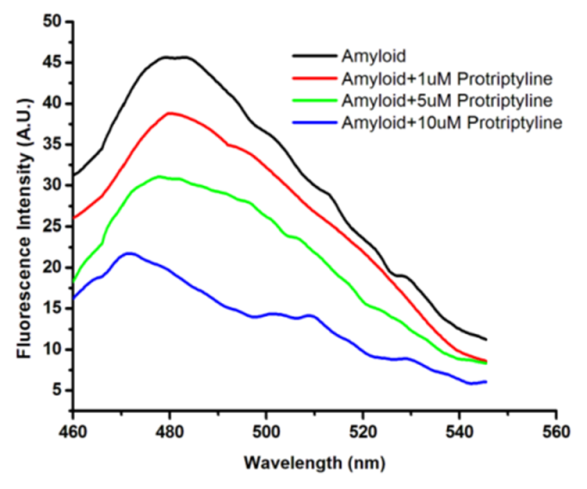

Supplement: Figure S1 — Thioflavin T Assay. Protriptyline causes concentration dependent decrease in Aβ aggregation. (DOCX) [file pone.0105196.s001.docx]
