# Supplementary material for: Molecular Investigations of Protriptyline as a Multi-Target Directed Ligand in Alzheimer's Disease
Source: PLoS One. 2014 Aug 20;9(8):e105196. doi: 10.1371/journal.pone.0105196 (PMC4139341; doi:10.1371/journal.pone.0105196)

**Supplementary Figure. S2. A.** Snapshot of drug binding with Aβ monomer. Central hydrophobic core are in line representation **B.** Comparison of residue wise percentage of helix from unbound and ligand bound Aβ monomer simulated trajectory.


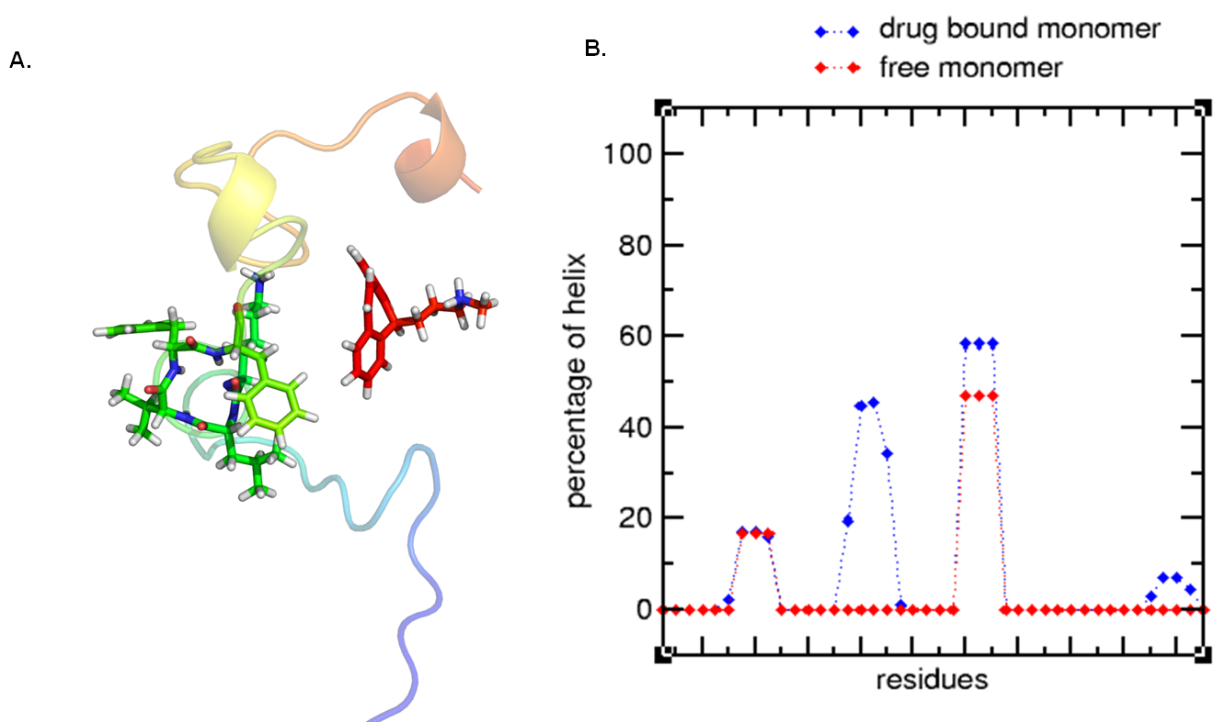

Supplement: Figure S2 — A. Snapshot of drug binding with Aβ monomer. Central hydrophobic core are in line representation B. Comparison of residue wise percentage of helix from unbound and ligand bound Aβ monomer simulated trajectory. (DOCX) [file pone.0105196.s002.docx]
