# Supplementary material for: Molecular Investigations of Protriptyline as a Multi-Target Directed Ligand in Alzheimer's Disease
Source: PLoS One. 2014 Aug 20;9(8):e105196. doi: 10.1371/journal.pone.0105196 (PMC4139341; doi:10.1371/journal.pone.0105196)

**Supplementary Figure. S3. A.** Root mean square deviation (RMSD) of active site regions of β-secretase in unbound (red) and ligand bound (blue) simulated trajectory **B**. Comparison of residue wise percentage of beta from unbound (red) and ligand bound (blue) simulated trajectory.


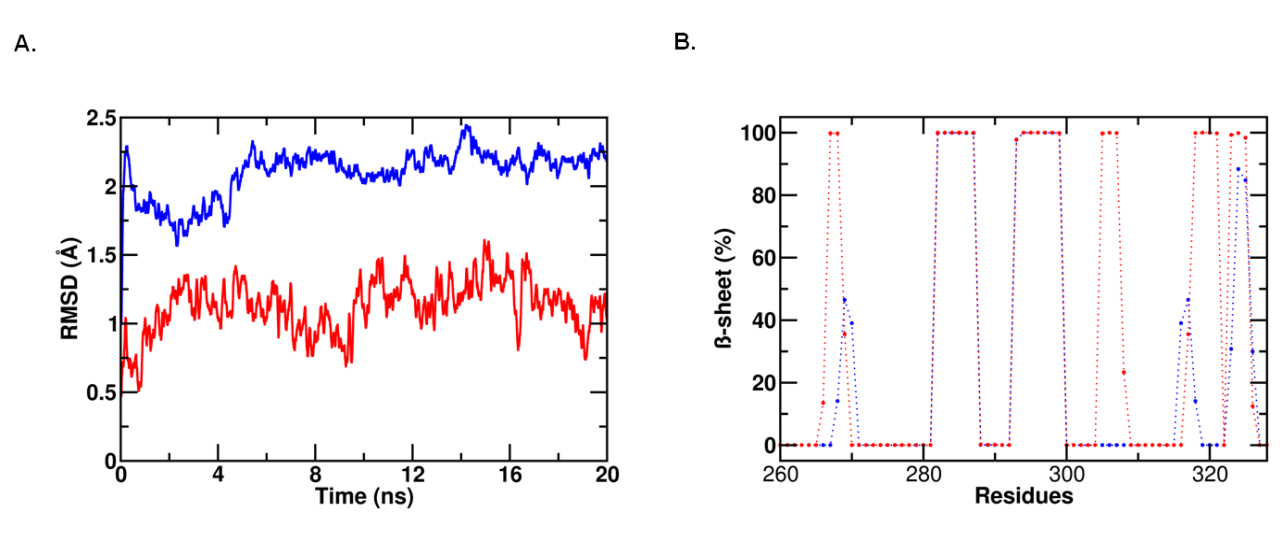

Supplement: Figure S3 — A. Root mean square deviation (RMSD) of active site regions of β-secretase in unbound (red) and ligand bound (blue) simulated trajectory. B. Comparison of residue wise percentage of beta from unbound (red) and ligand bound (blue) simulated trajectory. (DOCX) [file pone.0105196.s003.docx]
