# Supplementary material for: Molecular Investigations of Protriptyline as a Multi-Target Directed Ligand in Alzheimer's Disease
Source: PLoS One. 2014 Aug 20;9(8):e105196. doi: 10.1371/journal.pone.0105196 (PMC4139341; doi:10.1371/journal.pone.0105196)

**Supplementary Figure. S4. Insulin glycation inhibition assay. A.** Control insulin **B.** Glycated insulin **C.** glycation inhibition in presence of 500 μM and **D.** 1000 μM protriptyline. These spectra were acquired on a positive reflector mode by MALDI-TOF-MS. Glycated peaks are shown by black arrow


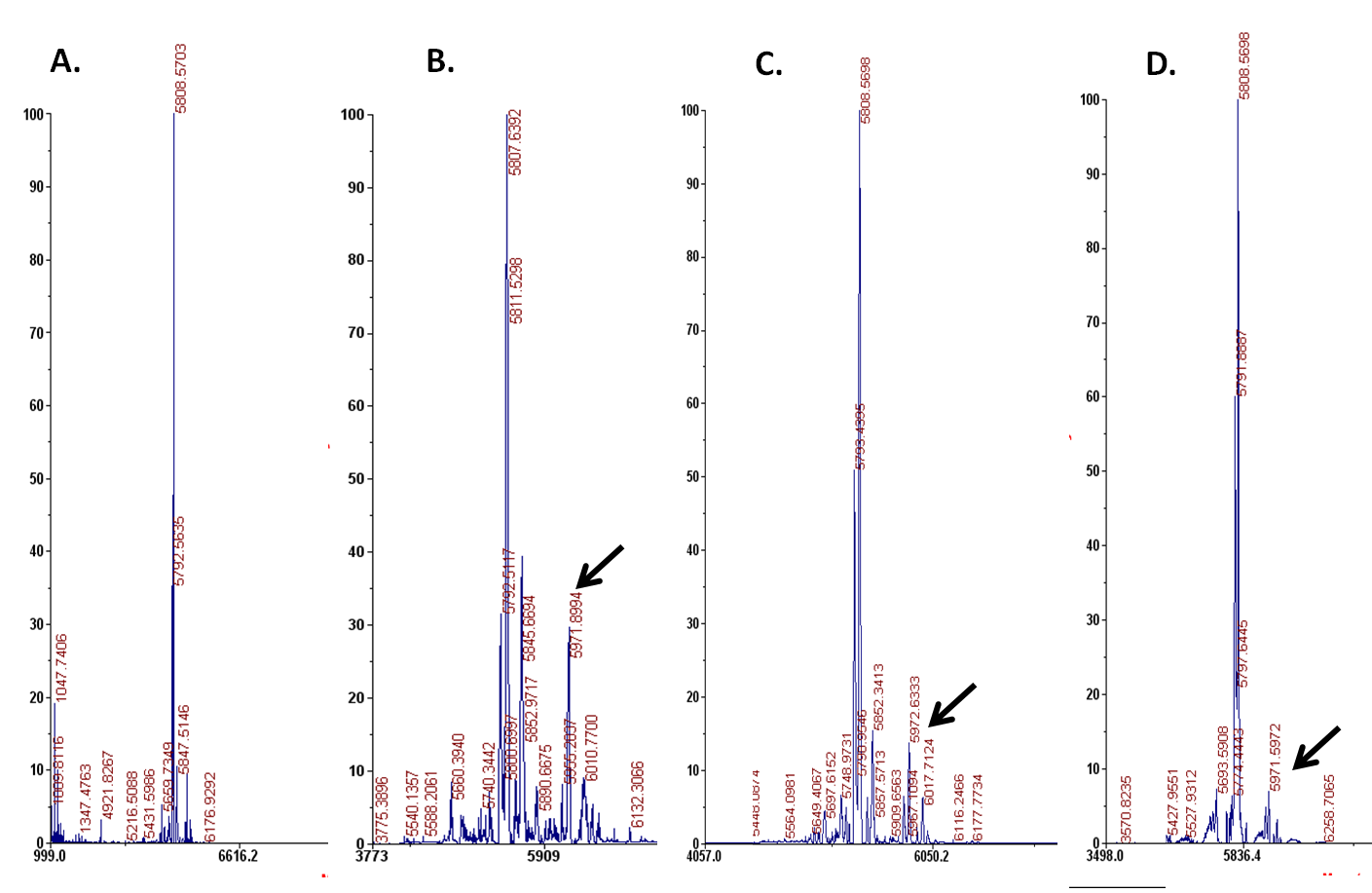

Supplement: Figure S4 — A. Control insulin B. Glycated insulin C. glycation inhibition in presence of 500 µM and D. 1000 µM protriptyline. These spectra were acquired on a positive reflector mode by MALDI-TOF-MS. Glycated peaks are shown by black arrow. (DOCX) [file pone.0105196.s004.docx]
