# Supplementary material for: Molecular Investigations of Protriptyline as a Multi-Target Directed Ligand in Alzheimer's Disease
Source: PLoS One. 2014 Aug 20;9(8):e105196. doi: 10.1371/journal.pone.0105196 (PMC4139341; doi:10.1371/journal.pone.0105196)

**Supplementary Figure. S5. BSA (Bovine Serum Albumin) glycation inhibition assay.** Glycation inhibition of BSA was studied by **A.** Measurement of AGE fluorescence , % glycation inhibition was plotted and **B.** Thioflavin T fluorescence assay, concentration dependent decrease in thioflavin T fluorescence was observed.


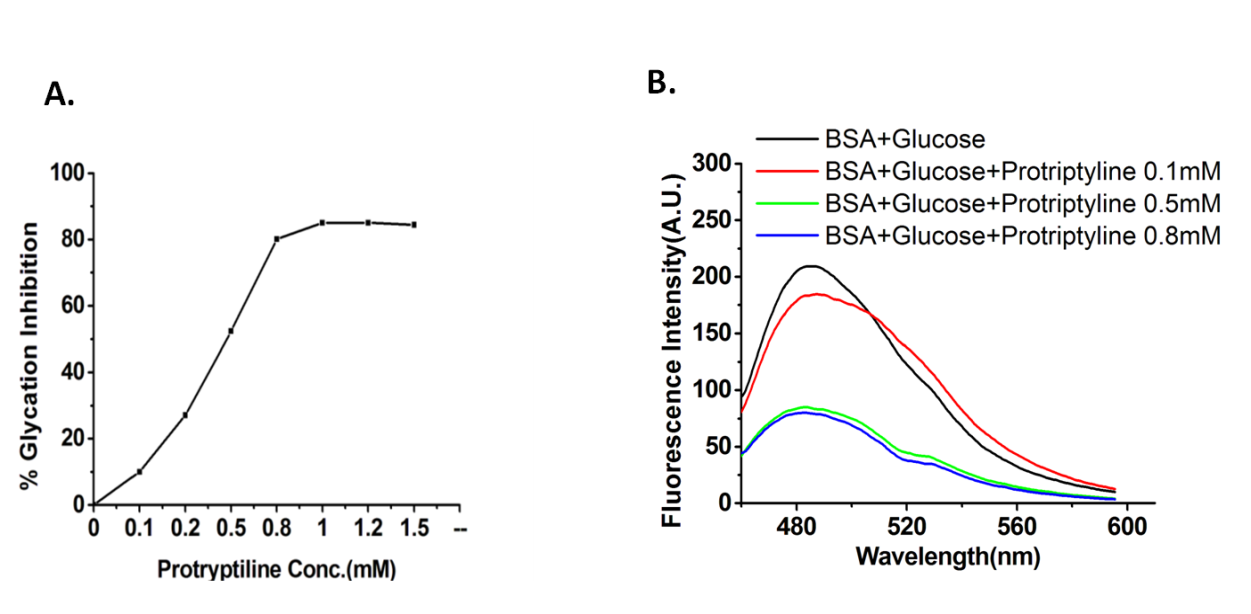

Supplement: Figure S5 — BSA (Bovine Serum Albumin) glycation inhibition assay. Glycation inhibition of BSA was studied by A. Measurement of AGE fluorescence, % glycation inhibition was plotted and B. Thioflavin T fluorescence assay, concentration dependent decrease in thioflavin T fluorescence was observed. (DOCX) [file pone.0105196.s005.docx]
