# Supplementary material for: Molecular Investigations of Protriptyline as a Multi-Target Directed Ligand in Alzheimer's Disease
Source: PLoS One. 2014 Aug 20;9(8):e105196. doi: 10.1371/journal.pone.0105196 (PMC4139341; doi:10.1371/journal.pone.0105196)
Supplement: Table S1 — Acetylcholinesterase-protriptyline interaction energy calculations. Interaction energy of ligand with active site residues averaged over last 20 ns of all simulated trajectories. Interaction strengths are in kcal mol-1 unit. Standard deviations are provided within braces. (DOCX) [file pone.0105196.s006.docx]

**Supplementary Tabel S1. Acetylcholinesterase-protriptyline interaction energy calculations.** Interaction energy of ligand with active site residues averaged over last 20 ns of all simulated trajectories. Interaction strengths are in kcal mol^-1^ unit. Standard deviations are provided within braces.

|  | **Cataytic/Esteraticsubsite** | | | **Anionic subsite** | | |
| --- | --- | --- | --- | --- | --- | --- |
|  | His^443^ | Glu^330^ | Ser^199^ | Trp^82^ | Glu^198^ | Tyr^333^ |
| **Total bindingenergy** | -13.0(4.0) | -5.9(2.9) | 0.5 (0.9) | -7.2(2.7) | -21.2(8.0) | -2.8(1.2) |
| **Electrostatic energy** | -9.3 (5.0) | -5.5 (2.8) | 0.6 (0.9) | -4.6 (2.0) | -20.9 (7.9) | 0.3 (0.6) |
| **vDW energy** | -3.3 (1.2) | -0.4 (0.3) | -0.05(0.03) | -2.6(1.6) | -0.2 (0.1) | -3.0 (1.0) |
