# Supplementary material for: Molecular Investigations of Protriptyline as a Multi-Target Directed Ligand in Alzheimer's Disease
Source: PLoS One. 2014 Aug 20;9(8):e105196. doi: 10.1371/journal.pone.0105196 (PMC4139341; doi:10.1371/journal.pone.0105196)
Supplement: Table S2 — Inter-residue distance between active site of AChE residues averaged over last 20 ns of free (vertical) and ligand bound (horizontal) simulated trajectories. Distances are in Å unit. Standard deviations are provided within braces. (DOCX) [file pone.0105196.s007.docx]

**Supplemenatry Table S2.** Interesidue distance between active site of AChE residues averaged over last 20 ns of free (vertical) and ligand bound (horizontal) simulated trajectories. Distances are in Å unit. Standard deviations are provided within braces.

|  | **Trp82** | **Glu198** | **Tyr333** | **Ser199** | **Glu330** | **His443** |
| --- | --- | --- | --- | --- | --- | --- |
| **Trp82** | - | 12.4 (0.5) | 14.9 (1.03) | 14.9 (0.6) | 16.0 (0.6) | 5.9 (0.6) |
| **Glu198** | 12.8 (0.4) | - | 15.8 (0.7) | 4.1 (0.3) | 9.8 (0.4) | 9.6 (0.8) |
| **Tyr333** | 14.0 (0.8) | 14.1 (1.0) | - | 14.5 (0.5) | 9.6 (0.5) | 11.1 (0.8) |
| **Ser199** | 12.7 (0.4) | 3.8 (0.1) | 12.2 (1.1) | - | 8.4 (0.4) | 11.5 (0.8) |
| **Glu330** | 17.5 (0.6) | 9.9 (0.4) | 8.9 (1.1) | 8.8 (0.4) | - | 10.5 (0.9) |
| **His443** | 6.5 (0.3) | 8.0 (0.3) | 9.5 (0.8) | 7.8 (0.3) | 10.3 (0.8) | - |
