# Supplementary material for: Molecular Investigations of Protriptyline as a Multi-Target Directed Ligand in Alzheimer's Disease
Source: PLoS One. 2014 Aug 20;9(8):e105196. doi: 10.1371/journal.pone.0105196 (PMC4139341; doi:10.1371/journal.pone.0105196)
Supplement: Method S1 — Inhibition of Insulin and BSA glycation. (DOCX) [file pone.0105196.s008.docx]

**Supplementary Method S1.**

**Inhibition of Insulin and BSA glycation.** 1.5mg/ml insulin and 0.1 M glucose were incubated with various concentrations of protryptiline in 10 mM PBS, pH 7.0 for 7 days. These samples were further used to study glycation inhibition by MALDI. BSA glycation reaction was performed in a similar manner with 1mg/ml BSA concentration and inhibition was studied by AGE fluorescence and Thioflavin T assay ^1^.

MALDI-TOF-MS based *in vitro* insulin glycation inhibition assay showed the glycated insulin peak, marked by an arrow. The intensity of glycated peak was observed to be reduced with increasing protriptyline concentrations. Similarly, inhibition of BSA glycation was proved by AGE fluorescence and Thioflavin T fluorescence. Concentration dependent decrease in glycation was observed.

**REFERENCE**

1. [Golegaonkar SB](http://www.ncbi.nlm.nih.gov/pubmed?term=Golegaonkar%20SB%5BAuthor%5D&cauthor=true&cauthor_uid=20212331), [Bhonsle HS](http://www.ncbi.nlm.nih.gov/pubmed?term=Bhonsle%20HS%5BAuthor%5D&cauthor=true&cauthor_uid=20212331), [Boppana R](http://www.ncbi.nlm.nih.gov/pubmed?term=Boppana%20R%5BAuthor%5D&cauthor=true&cauthor_uid=20212331), [Kulkarni MJ](http://www.ncbi.nlm.nih.gov/pubmed?term=Kulkarni%20MJ%5BAuthor%5D&cauthor=true&cauthor_uid=20212331) (2010) Discovery of rifampicin as a new anti-glycating compound by matrix-assisted laser desorption/ionization mass spectrometry-based insulin glycation assay. Eur J Mass Spectrom 16(2): 221-226
