# Supplementary material for: Molecular Investigations of Protriptyline as a Multi-Target Directed Ligand in Alzheimer's Disease
Source: PLoS One. 2014 Aug 20;9(8):e105196. doi: 10.1371/journal.pone.0105196 (PMC4139341; doi:10.1371/journal.pone.0105196)
Supplement: Method S2 — System Setup for Molecular Dynamics Simulation. (DOCX) [file pone.0105196.s009.docx]

**Supplementary Method S2.**

**System Setup for Molecular Dynamics Simulation:**

**AChE.** Initial coordinates for the AChE molecules were derived from the X-ray crystallographic structure of the enzyme complexed with fasciculin-II^1^ reported in the PDB database (PDB ID 1B41). Fasciculin was deleted from the structure, and the missing residues were added with the *Modeller* software. Protriptyline was docked with the catalytic site residues (Ser199, Glu330, His443) using Autodock. The resultant system was immersed in a box of equilibrated TIP3P water molecules^2^, maintaining a minimum distance of 14 Å from the box edge to each atom. Seven sodium counter ions were added at random positions within the simulation box.

**Aβ.** Amyloid β (Aβ) is an intrinsically unstructured peptide whose high aggregation propensity makes it forbiddingly difficult to report the monomeric structure with purely experimental means in fully aqueous environment. However, NMR studies have reported the monomeric structure in mixtures of organic solvent and water ^3^. Our initial structure was generated by heating and quenching full length Aβ (PDB ID 1ZOQ) in water. Heating this structure in 100% water for 2 ns at 600 K resulted in a random coil state, which was quenched at 310 K and four independent 100 ns trajectories were generated. Two of the trajectories resulted in monomeric structures with significant anti-parallel beta sheet content in the C-terminal region and marginal helicity in the N-terminal region, in excellent agreement with recent reports of the important conformations in the Aβ ensemble ^4, 5^. Five protriptyline molecules were placed randomly in the vicinity of the monomeric structure thus obtained, and the resilting system placed in an equilibrated box of TIP3P water in a manner similar to AChE. The protriptyline-Aβ dimer complex was obtained by adding an incoming monomer at a distance of 25 Å from the center of mass of the protriptyline bound monomeric state. Chloride counter ions were added in each case to maintain charge neutrality. The free monomer and dimer complexes were simulated separately with equivalent protocols.

**BACE1.** The initial co-ordinates of human β-secretase (BACE-1) were obtained from PDB ID 2HM1, which is a 2.2 Å resolution crystallographic structure of the protein bound with hydroxyethylamine ^6^. The latter was removed, missing residues added with *Modeller*, and the active residues Asp32 and Asp228 of the resultant structure was docked with protriptyline. Previous studies point out that Asp228 of the active site of beta secretase is protonated ^7^. Asp protonation was done accordingly in our setup. The His residues were protonated in order to mimic the experimental conditions of pH 5.0, and the system was immersed in an equilibrated box of TIP3P water as the other systems. Three sodium counter ions were added at random positions.

**Simulation Analyses.**

**Asphericity (I_γ_).** The minimum (*I_min_*) and the maximum (*I_max_*) values of the principle moments of intertia of the protein were first calculated for every snapshot using the VMD software ^8^, and the asphericity was obtained as,

$$I_{\gamma}=1-\frac{I_{\min}}{I_{\max}}$$

For a perfectly spherical object, *I_min_* and *I_max_* are equal, leading to an *I_γ_* value of 0.0 Thus, higher values of *I_γ_* denote deviations from spherical gemoetry, or greater asphericity.

**Inter-residue contact maps.** A pair of residues belonging to different peptide units were considered to make contact if their centers of mass approached within 7.0 Å of each other. The inter-residue contact maps were obtained by calculating the contact probabilities of each inter-protein residue pair from the simulated snapshots.

**Secondary Structure*.*** Secondary structures reported are calculated within the VMD package using the algorithm STRIDE ^9^.

**REFERENCES**

1. Kryger G, Harel M, Giles K, Toker L, Velan B, et al. (2000) Structures of recombinant native and E202Q mutant human acetylcholinesterase complexed with the snake-venom toxin fasciculin-II. Acta Crystallogr. 56: 1385-1394.
2. Jorgensen WL, Chandrasekhar J, Madura JD, Impey RW, Klein ML (1983) Comparison of Simple Potential Functions for Simulating Liquid Water. J. Chem. Phys. 79: 926–935.
3. Tomaselli S, Esposito V, Vangone P, van Nuland NAJ, Bonvin AMJJ, et al. (2006) The alpha-to-beta conformational transition of Alzheimer's Abeta-(1-42) peptide in aqueous media is reversible: a step by step conformational analysis suggests the location of beta conformation seeding. ChemBioChem 7: 257–267.
4. Lin Y, Bowman GR, Beauchamp KA, Pande VS (2012) Investigating how peptide length and a pathogenic mutation modify the structural ensemble of amyloid beta monomer. Biophysical Journal 102: 315–324.
5. Sgourakis NG, Yan Y, McCallum SA, Wang C, Garcia AE (2007) The Alzheimer's peptides Abeta40 and 42 adopt distinct conformations in water: a combined MD / NMR study. J. Mol. Biol. 368: 1448–1457.
6. Freskos JN, Fobian YM, Benson TE, Moon JB, Bienkowski MJ, et al. (2007) Design of potent inhibitors of human beta-secretase. Part 2. Bioorg.Med.Chem.Lett. 17: 78-81.
7. Xiong B, Huang X, Shen L, Shen J, Luo X, et al. (2004) Conformational flexibility of beta-secretase: molecular dynamics simulation and essential dynamics analysis. Acta Pharmacol Sin 25: 705-713.
8. Humphrey W, Dalke A, Schulten K (1996) VMD: visual molecular dynamics. J. Mol. Graph. 14: 33-38.
9. Frishman D, Argos P (1995) Knowledge-based protein secondary structure assignment. Proteins: Struct. Funct. Genet. 23: 566–579.
